# Primary epidermoid carcinoma of the breast presenting as a breast abscess and sepsis

**DOI:** 10.1590/S1516-31802011000600009

**Published:** 2011-12-01

**Authors:** Andrea Pires Damin, Fernanda Costa Nascimento, João Batista Andreola, Talita Haubert Cerutti, Adriana Roehe, Daniel Carvalho Damin

**Affiliations:** I MD, PhD. Attending Physician, Division of Breast Surgery, Hospital Fêmina, Porto Alegre, Rio Grande do Sul, Brazil.; II MD. Attending Physician, Division of Breast Surgery, Hospital Fêmina, Porto Alegre, Rio Grande do Sul, Brazil.; III MD, PhD. Adjunct Professor, Department of Pathology, Universidade Federal de Ciências da Saúde, Porto Alegre, Rio Grande do Sul, Brazil; IV MD, PhD. Adjunct Professor, Department of Surgery, Universidade Federal do Rio Grande do Sul, Porto Alegre, Rio Grande do Sul, Brazil.

**Keywords:** Breast neoplasms, Carcinoma, squamous cell, Abscess, Sepsis, Mastectomy, Neoplasias da mama, Carcinoma de células escamosas, Abscesso, Sepse, Mastectomia

## Abstract

**CONTEXT:**

Squamous cell carcinoma (SCC) of the breast is an extremely rare form of cancer, accounting for approximately 0.04% of all malignant breast tumors. To date, only a limited number of cases of SCC of the breast have been reported, and most of them presented like the usual breast carcinomas.

**CASE REPORT:**

A 39-year-old woman presented with a large breast abscess and signs of sepsis. After surgical debridement of the lesion, histopathological examination of the abscess capsule revealed the presence of SCC of the breast. The definitive treatment for the tumor consisted of modified radical mastectomy with resection of the residual lesion in the right breast.

**CONCLUSIONS:**

This unusual case illustrates how an apparently benign disorder such as a breast abscess might be related to a clinically occult malignancy. A review of the literature on SCC of the breast is presented.

## RESUMO

**CONTEXTO:**

O carcinoma de células escamosas da mama é um câncer extremamente raro, representando cerca de 0,04% dos tumores malignos da mama. Até o momento, apenas um número limitado de casos da doença foi relatado, a maioria se apresentando clinicamente como um carcinoma de mama usual.

**RELATO DE CASO:**

Paciente feminina de 39 anos de idade se apresentando com grande abscesso de mama e sinais de sepse. Após debridamento cirúrgico da lesão, o exame histopatológico da cápsula do abscesso revelou a presença de carcinoma de células escamosas da mama. O tratamento definitivo do tumor foi uma mastectomia radical modificada com ressecção da lesão residual na mama direita.

**CONCLUSÃO:**

O presente caso, de apresentação incomum, demonstra como uma lesão aparentemente benigna, como um abscesso mamário, pode estar relacionada a uma neoplasia maligna oculta. Uma revisão da literatura a respeito do carcinoma de células escamosas de mama é apresentada.

## INTRODUCTION

Primary squamous cell carcinoma (SCC) of the breast is an exceedingly rare disease, accounting for approximately 0.04% of all breast malignancies.^1^ Its diagnosis is established when no other primary malignancy can be found in the resected breast specimen; presence of a breast metastasis from another primary tumor is ruled out; and the tumor does not arise from the breast skin. To date, only a limited number of cases of SCC of the breast have been reported, and most of them presented like the usual breast carcinoma.^2,3^ In this paper, we report on a case of SCC of the breast that atypically presented as a large and life-threatening breast abscess.

## CASE REPORT

**Figure 1:**
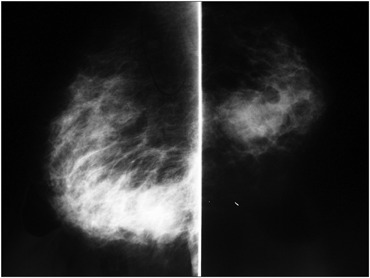
Radiological appearance of the lesion in the mediolateral and craniocaudal mammographic views.

**Figure 2:**
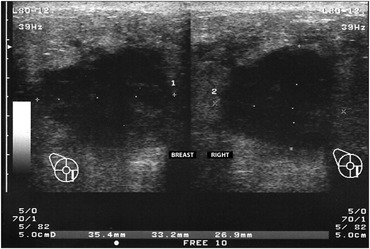
Breast ultrasound showing an irregular lesion with a central cavity containing liquid material.

A 39-year-old woman without any history of trauma or breast disease presented at Hospital Fêmina with a progressive painful swelling of the right breast that she had first noted one month earlier. Upon physical examination, she presented several signs of severe infection such as high fever, chills, unstable mental state and lethargy. Breast examination revealed a large painful mass involving both lower quadrants of the right breast. Laboratory tests showed anemia (hemoglobin 8.5 g/dl) and a high white blood cell count (12,000/mm) with a marked left shift. A mammogram demonstrated an irregular abnormality measuring 2.5 × 3.3 × 2.6 cm in the right breast, which was classified as BIRADS 5 (Figure 1). Breast ultrasound showed a 3.5 cm irregular mass containing a fluid accumulation (Figure 2). An ultrasound-guided core biopsy was then performed, and this revealed atypical ductal hyperplasia with accentuated pleomorphism.

After admission to the hospital, the patient suffered rapid deterioration of her general clinical condition. Within 24 hours, she showed overt signs of sepsis, despite the use of systemic broad-spectrum antibiotics. Since no other source of infection was identified, she was subjected to an urgent exploration of the right breast. During the procedure, a large mass extending from the lower mammary quadrants up to the retro-areolar region was identified. The appearance of the lesion was atypical, with imprecise limits and a large central cavity containing necrotic material. A biopsy of the capsule of this lesion was obtained. After surgical debridement of this material, the patient started to present improvements in all the inflammatory signs and symptoms.

Histopathological analysis on the surgical biopsy revealed an undifferentiated carcinoma with extensive areas of necrosis. Additionally, immunohistochemical analysis demonstrated high expression of cytokeratin (34bE12) within the tissues, which was compatible with a diagnosis of epidermoid carcinoma of the breast. The breast tumor profile was negative for estrogen receptors, progesterone receptors and HER2/neu overexpression.

**Figure 3:**
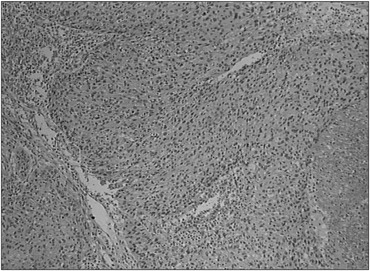
Histological features of the surgical specimen showing epidermoid carcinoma (high-power field).

In view of the pathological findings, the patient underwent modified radical mastectomy with resection of the residual lesion in the right breast three weeks after the first surgery. Histopathological analysis on the surgical specimen demonstrated a poorly differentiated epidermoid carcinoma, which did not involve the breast skin (Figure 3). No metastases were detected in the 13 axillary lymph nodes that were resected. After postoperative recovery, the patient was referred for complementary radiotherapy to the chest wall. She did not receive adjuvant chemotherapy. She remains free of disease two years after the definitive surgical treatment.

## DISCUSSION

Primary squamous cell carcinoma of the breast is a very rare disease. Its diagnosis is established when the malignant cells are all of squamous cell type, it does not arise from the skin of the breast and there is no other primary SCC anywhere else in the body.^4^

Although these tumors most frequently present like the usual breast carcinoma, SCC of the breast may, on very rare occasions, present as a breast abscess.^5^ A systematic survey of indexed articles using the terms "epidermoid carcinoma of the breast" and "abscess" and using the terms "squamous cell carcinoma", "breast" and "abscess" in the Lilacs (Literatura Latino-Americana e do Caribe em Ciências da Saúde), Embase (Excerpta Medica Database), Scirus, SciELO, Medline and Cochrane Library databases and MeSH (Medical Subject Headings) revealed that only nine articles have been published on this topic to this date. All of these papers were found in Medline (Table 1).^5-13^ A less specific survey using the terms "epidermoid carcinoma" and "breast (MeSH terms)" and using the terms "squamous cell carcinoma" and "breast (MeSH terms)" was also conducted. However, this proved to be less productive, since only five articles written in English since 1980 could be found.^14-19^ None of these articles (all found in Medline) describe any cases of epidermoid carcinoma of the breast presenting as abscess or sepsis.

**Table 1:** Results from our reviews of medical databases using descriptors for the main clinical findings observed in our patient

Data base	Search strategy	Results*
PubMed	"epidermoid carcinoma of the breast" AND "abscess"	8 case reports^5-12^ No reviews of the literature
	"epidermoid carcinoma of the breast" AND "sepsis"	No case reports or reviews of the literature
	"squamous cell carcinoma of the breast "AND "abscess"	9 case reports^5-13^ No reviews of the literature
	"squamous cell carcinoma of the breast" AND "sepsis"	No case reports or reviews of the literature

* Using the same search strategy in the Cochrane Library, SciELO, EMBASE, MeSH and Lilacs databases, no results were found. A search in the Scirus database revealed the same articles as found in PubMed.

It is important to consider SCC in the differential diagnosis of breast abscess when there is no initial clinical response to drainage of a breast abscess or to administration of broad-spectrum antibiotics. Wrightson et al. described three cases of SCC of the breast. One of them presented as a breast abscess that was initially managed by surgical drainage and debridement of necrotic material. Similarly to what happened in our case, the definitive treatment for their patient required that mastectomy should be performed.^6^

It is important to highlight that SCCs of the breast should be distinguished from mixed tumors in which some areas containing squamous cells can be found within primary adenocarcinoma of the breast. It is also essential to rule out the presence of SCC elsewhere in the body, which might represent the actual primary tumor from where the breast SCC is derived (metastatic breast involvement).^1,4^

SCC of the breast is a difficult tumor to diagnose through the subsidiary imaging examinations commonly used. There are no typical findings on the mammogram. Although ultrasound may show the presence of a complicated cyst or an inflammatory process, these tumors have not been reported as showing any specific characteristics. A biopsy should be always obtained, as it definitively confirms the diagnosis of SCC of the breast.^20,21^ In most of the reported cases, the diagnosis was based solely on the histopathological findings. Immunohistochemical analysis showing tumor cells positively stained for cytokeratin was used by some authors to further support the pathological findings.^22^ In the present case, immunohistochemistry was positive for cytokeratin, but negative for hormone receptors. According to Siegelmann-Danieli et al., estrogen and progesterone receptors are negative in more than 90% of pure squamous cell carcinomas of the breast.^23^

The treatment for SCC of the breast is similar to that of other malignant breast tumors. Although a conservative approach to the breast can be used, many of these patients already present locally advanced disease at diagnosis, thus precluding breast conservation. In addition, it has been demonstrated that auxiliary dissection is a fundamental part of the treatment.^21,24,25^ Because of the rarity of this cancer, only limited data are available on the role of chemotherapy.^4^ The therapeutic combination most commonly used is 5-fluorouracil and cisplatin, and some degree of success from this has been reported.^4,26,27^ The combination of docetaxel and doxorubicin has been reported to be an alternative in some cases.^17,26-29^ Complete clinical and pathological responses to platinum agent-based regimens have been described in patients with either systemic metastases^27^ or locally advanced disease.^17^ The role of radiation has been described as unclear in many studies. Although SCC is generally radiosensitive, locoregional relapse occurs frequently and may even involve the irradiated field.^20,21,30^ Since previous cases of SCC have predominantly been hormone receptor-negative, there only seems to be a limited role for hormonal therapy in this type of cancer.^2,20,27^

## CONCLUSION

We reported an extremely rare case of SCC of the breast atypically presenting as a breast abscess that rapidly progressed to overt sepsis. This case illustrates that an apparently benign disorder such as a breast abscess might occasionally be related to a clinically occult malignancy. Complicated cysts and breast abscesses should always be evaluated through histopathological examination.
